# Typho-Malarial Fever

**Published:** 1896-12

**Authors:** I. P. Sessions

**Affiliations:** Rockdale, Tex.


For the Texas Medical Journal.

### TYPHO-MALARIAL FEVER.

BY DR. I. P. SESSIONS, ROCKDALE, TEX.

Read before Brazos Valley Medical Association held at Bryan, Texas, November 10, 11, 1896.

IN SPEAKING on this subject, I refer especially to the fever so prevalent in the Southern States, and warm climates in general. The pathology of this disease is by no means clearly defined; and until more careful and accurate post-mortems have been made, and until the microscope has yielded to us the bacteriological factor in the disease, then, and not till then, will we cease to be on debatable grounds concerning the nature of "so-called" typho-malarial fever.

The theories in our profession, with regard to its nature, are about as follows:

1st. It is a complication of typhoid fever with malarial fever.

2d. It is a hybrid disease, formed by the coalition of typhoid fever with malarial fever.

3d. It is a remittent form of malarial fever.

4th. It is a true typhoid fever modified in form.

5th. It is a separate and distinct type of specific fever, assimilating both typhoid fever and malarial fever, but originating from a different cause, and at the same time having symptoms peculiar to itself.

If you answer the first theory in the affirmative, then will we have the curious anomaly of the virulence of one disease being mitigated by the addition of another.

We almost daily come in contact with cases in which one disease is a complication of others; in none of which cases is the severity of the symptoms lessoned, but more frequently increased.

The alimentary canal is a common seat of inflammatory complications. A gastritis becoming gastro-enteritis; an enteritis becoming an entero-colitis, etc.

We may have scarlet fever complicated by diphtheria; or meningitis complicating small-pox. Bright's disease may complicate a hepatitis, and gonorrhœa may complicate syphilis. But I have my first case to see where one disease complicating another lessens the severity of the symptoms of the original disease. Is it not strange, then, that malarial fever has this wonderful power? If this is true, we have found a very valuable remedy for typhoid fever.

The prognosis of "so-called" typho-malaria is so favorable as compared with typical typhoid, that it can hardly be considered. Hence, when we have a case of typhoid let us treat it by inoculating it with the malarial plasmodium or send it to the swamps as soon as possible. How would it do to set up a sanitarium or health resort in the Brazos bottom, exclusively for the use of typhoid patients?

As an argument in favor of the first theory, you may say that there are very few cases of typical typhoid fever in malarial districts. To this, I will answer, that there is also very little scarlet fever and diphtheria in these same localities. So, according to the same reasoning, I may say that malaria is a great panacea for these diseases.

Now, if this is a complication, and it is true that these two diseases have such an affinity for each other, is it not strange that we do sometimes have severe epidemics of typical typhoid in malarial districts, in which cases the malarial poison seems to exert no influence whatever over the disease? And again, since we so seldom have a case of typical typhoid either before, during or after an epidemic of typho-malaria, and since we acknowledge that typhoid is caused by the typhoid bacillus, then I would ask—from whence does this peculiar bacillus come, and where does it go? and besides, no microscopist has, so far, been able to find the baccillus of typhoid in "so-called" typho-malarial fever.

2. Is it a hybrid disease?

The arguments above will practically answer this, namely; we never have a history of typhoid coming in contact with malarial fever and typho-malaria being the result of the union.

How many hybrid diseases can you enumerate? If this is one, it is the only one known to the medical world; and besides, its symptoms, as I will endeavor to show, are so unlike typhoid on the one hand, and malaria on the other, that it may, to all intents and purposes, be considered a separate disease.

3. Is it a remittent form of malarial fever? While we have remissions in this disease, as we do in any form of protracted fever, yet the remissions do not appear with enough regularity to stamp it as a remittent malaria. I believe that this disease is often complicated by malaria, for we sometimes find the malarial plasmodium, but it lacks a great deal of being cured when the plasmodium has disappeared by the judicious use of treatment ordinarily used for malarial fever.

Typho-malaria is not amenable to any known anti-malarial treatment; and even quinine, the great sheet anchor in all forms of malaria, frequently aggravates its symptoms.

4. Is it a true typhoid, modified in form?

This is the opinion held by a great majority of the members of our profession. I have seen an article in a recent journal claiming that this is the case, and at the same time the author says he has long since quit trying to diagnose the disease by the symptoms laid down in our text-books on typhoid. Well may he have made this resolution, for when we come to carefully compare the symptoms of the two diseases, we will be surprised that the points of difference have been so long overlooked. Before we begin the analysis of this theory, let us understand what is meant by a modified disease. I do not think it is one, in which the symptoms differ from the original type, but a case in which the symptoms are less severe. A slight diarrhœa may be a modified symptom of dysentery but constipation is not.

Varioloid, which has nearly all of the symptoms of confluent small-pox, but less severe in character, is my idea of a disease modified in form.

While I grant you we may and do have epidemics of true typhoid in this country, yet I do not think that "so-called" typho-malaria is a modified typhoid. I know that the morbid anatomy of this disease very closely resembles that of typhoid, but not more so than the lesion of many kindred diseases resembles each other. Loomis says, that the intestinal changes resemble those of typhoid on the one hand, and remittent fever on the other, yet its clinical differences are sufficient to distinguish it from both, and to stamp it as a distinct type of fever.

Let us compare some of the most frequent and common symptoms of typhoid as recorded by our text-books, and as it is seen in epidemics of undisputed typhoid, with some of the symptoms of typho-malaria.

The duration of typhoid is about four weeks; the duration of typho-malaria is from 14 to 28 days.

In the beginning of typhoid there is a gradual rise of temperature (rarely beginning with a chill) to the sixth or eighth day, with morning remissions and evening exacerbations. Then comes a period when the morning and evening temperature remains nearly the same; after which the remissions begin, and the evening temperature gradually gets lower, to the end of the disease.

Dr. Pepper says, that a temperature of 104° during the first or second day will exclude a diagnosis of typhoid. Typho-malaria is usually ushered in by a chill, or chilly sensations, and the temperature reaches its maximum on the first, second or third day from the initiation. The temperature curve does not follow that of typhoid, but usually falls somewhat on the third or fourh day, followed by an irregular temperature to the end of the disease. I have not infrequently seen a temperature of 103° a day or two before the termination of the disease.

In the beginning of typhoid the face has an anxious expression, with eyes bright and pupils dilated; loss of strength occurs early.

In typho-malaria the facial expression is natural, and loss of muscular strength does not occur till late in the disease.

All of our text-books agree that wakefulness is a very frequent and common symptom of typhoid. In typho-malaria the reverse of this is true. I have very seldom been forced to give somnifacients in typho-malaria. The rose-colored spots of typhoid are never found in typho-malaria.

One of the most frequent and early symptoms of typhoid is some mental derangement, such as inability to fix thought, or partial loss of memory. Pepper says, that he relies on this one symptom, more than on all others, in his diagnosis. In typho-malaria the mind is clear, except in the latter stage of severe cases.

Delirium is common in typhoid, while it is very uncommon in typho-malaria. In typhoid persistent headache is the rule. In typho-malaria it is seldom seen, except during high fever. The typhoid condition, so frequently met with in typhoid, is rarely seen in typho-malaria.

The pulse rate in general is more frequent in typhoid than in typho-malaria.

I have seen a number of cases whose pulse rate from the beginning was below normal.

The one great diagnostic symptom of typhoid is diarrhœa; not only on account of the frequency of its occurrence, but also from its peculiar character. True, we sometimes have constipation in pure typhoid, but it is the exceptional case.

In typho-malaria constipation is the rule, and even when we do have a case with diarrhœa the stools are never like the peculiar discharges of typical typhoid.

Now, about the only symptoms that are most frequently common to the two diseases are as follows:

1. Tympanitis.
2. Iliac tenderness.
3. Gurgling on pressure in iliac region.
4. Hemorrhage from the bowels.
5. Enlarged liver and spleen.

Tympanitis is not limited to these diseases, but is found in others. It is much more marked in typhoid than in typho-malaria.

Iliac tenderness is only a result of the lesion of the two diseases, hence not a diagnostic symptom of either.

Gurgling is found more frequently in typhoid than in typho-malaria.

Hemorrhage from the bowels is more frequent in typhoid than in typho-malaria, and besides, it is also seen in other diseases with intestinal lesions.

The liver and spleen, especially the latter, are much more enlarged in typho-malaria than in typhoid.

Now, I do not believe that the identity of the two diseases can be established by symptomatology; and while I grant that the lesion is very similar, yet, until the typhoid bacillus can be found in typho-malaria, I will have very strong grounds to doubt that the lesions are identical.

5. Is it a separate and distinct type of specific fever?

I think it has been conclusively shown that it is not a complication of typhoid with malaria, nor a hybrid, nor a remittent malaria, nor a modified typhoid; so, by the method of exclusion, I am persuaded that it is a separate disease, depending on neither typhoid nor malaria for its origin.

---

NOTE:—There are some who claim to have found the typhoid bacillus in typho-malarial patients, but the experiments have not been satisfactory, even to the experimentors; for they have not been able to reproduce the disease by inoculating healthy subjects with the bacillus found. And, besides, it is possible that the subjects in which the bacillus was found really died from typhoid, and not from typho-malaria.

